# Direct Comparison of Bayesian and Fermi Deconvolution Approaches for Myocardial Blood Flow Quantification: *In silico* and Clinical Validations

**DOI:** 10.3389/fphys.2021.483714

**Published:** 2021-04-12

**Authors:** Clément Daviller, Timothé Boutelier, Shivraman Giri, Hélène Ratiney, Marie-Pierre Jolly, Jean-Paul Vallée, Pierre Croisille, Magalie Viallon

**Affiliations:** ^1^Univ Lyon, INSA-Lyon, Université Claude Bernard Lyon 1, UJM-Saint Etienne, CNRS, Inserm, CREATIS, UMR 5220, U1294, Lyon, France; ^2^Department of Research and Innovation, Olea Medical, La Ciotat, France; ^3^Siemens Medical Solutions USA, Inc., Boston, MA, United States; ^4^Siemens Healthineers Princeton, Princeton, NJ, United States; ^5^Division of Radiology, Faculty of Medicine, Geneva University Hospitals, University of Geneva, Geneva, Switzerland; ^6^Department of Radiology, CHU de Saint-Etienne, University of Lyon, UJM-Saint-Etienne, Saint-Étienne, France

**Keywords:** myocardial perfusion quantification, cardiovascular magnetic resonance, Bayesian, perfusion-weighted imaging, heart disease, ischemic lesion, cardiovascular microcirculation, myocardial blood flow

## Abstract

Cardiac magnetic resonance myocardial perfusion imaging can detect coronary artery disease and is an alternative to single-photon emission computed tomography or positron emission tomography. However, the complex, non-linear MR signal and the lack of robust quantification of myocardial blood flow have hindered its widespread clinical application thus far. Recently, a new Bayesian approach was developed for brain imaging and evaluation of perfusion indexes (Kudo et al., 2014). In addition to providing accurate perfusion measurements, this probabilistic approach appears more robust than previous approaches, particularly due to its insensitivity to bolus arrival delays. We assessed the performance of this approach against a well-known and commonly deployed model-independent method based on the Fermi function for cardiac magnetic resonance myocardial perfusion imaging. The methods were first evaluated for accuracy and precision using a digital phantom to test them against the ground truth; next, they were applied in a group of coronary artery disease patients. The Bayesian method can be considered an appropriate model-independent method with which to estimate myocardial blood flow and delays. The digital phantom comprised a set of synthetic time-concentration curve combinations generated with a 2-compartment exchange model and a realistic combination of perfusion indexes, arterial input dynamics, noise and delays collected from the clinical dataset. The myocardial blood flow values estimated with the two methods showed an excellent correlation coefficient (*r*^2^ > 0.9) under all noise and delay conditions. The Bayesian approach showed excellent robustness to bolus arrival delays, with a similar performance to Fermi modeling when delays were considered. Delays were better estimated with the Bayesian approach than with Fermi modeling. An *in vivo* analysis of coronary artery disease patients revealed that the Bayesian approach had an excellent ability to distinguish between abnormal and normal myocardium. The Bayesian approach was able to discriminate not only flows but also delays with increased sensitivity by offering a clearly enlarged range of distribution for the physiologic parameters.

## Introduction

Coronary artery disease (CAD) and microvascular dysfunction are the major mechanisms leading to perfusion abnormalities. Microvascular dysfunction, which is responsible for true ischemic signs despite nearly normal coronary arteries, is a very challenging diagnosis. In CAD, clinical decision making relies on the relationship between symptoms and the degree of coronary lesions. In all these scenarios, cardiac magnetic resonance myocardial perfusion imaging has been proposed as an important detection tool and a gatekeeper for invasive diagnostic procedures and percutaneous coronary interventions.

The added value of truly quantitative measures of perfusion has been discussed in recent papers and avoids intrinsic subjectivity of visual perfusion assessment (Lee and Johnson, 2009; Gould et al., 2013). Measures of myocardial blood flow (MBF) not only contribute to unraveling the specific pathophysiological mechanisms underlying preclinical conditions but also provide a more reliable characterization of CAD burden (Knaapen, 2014).

Compared to single-photon emission computed tomography or positron emission tomography, cardiac magnetic resonance (CMR) has several advantages: no radiation exposure, no attenuation artifacts and a high spatial resolution. In multicenter trials, CMR has been validated against SPECT imaging with better results for the detection of CAD lesions (Greenwood et al., 2012; Schwitter et al., 2013). Furthermore, CMR demonstrated excellent accuracy against fractional flow reserve, the reference gold standard invasive method evaluating the stenosis-related decline in distal coronary pressure during maximum hyperemia (Li et al., 2014). Despite these results, routine CMR still has great potential for improvement, and several papers have already listed the difficulties of optimal quantification of perfusion parameters by CMR (Jerosch-Herold et al., 2004b; Cernicanu and Axel, 2006; Kellman and Arai, 2007; Gould, 2009; Lee and Johnson, 2009; Jerosch-Herold, 2010).

Briefly, absolute quantification of myocardial perfusion requires the integration of numerous technical stages from MR imaging to the measurement of hemodynamic parameters. Each step combines complex techniques to overcome constraints of cardiac and respiratory motions, coil inhomogeneity, non-linearity between MR signals and local contrast agent concentrations.

Regarding pure kinetic modeling, various methods have been proposed to determine regional MBF in myocardial sectors or pixelwise maps. These methods are commonly classified into two categories: parametric-indicator kinetic models and model-independent approaches. Compartment models describe the temporal evolution of contrast agent concentrations in distinct spaces with specific features. The model properties are adjusted to optimize the fitted tissue concentration-time curve C_*t*_(t). Depending on the expected level of accuracy, the chosen model may rely on an inflationary number of parameters requiring assumptions that are hardly sustainable, especially in pathophysiological conditions. Furthermore, the quantification of perfusion is an ill-posed inverse problem, and increasing the number of parameters in a model may lead to non-unique solutions that could be the source of considerable estimation errors (Jerosch-Herold et al., 1998). Often, it results in a difficult compromise between the accuracy of tissue behavior and the assumptions needed for a unique solution.

Model-independent approaches rely on the indicator dilution theory introduced by Zierler (1965); Pack and DiBella (2010). This principle considers a system composed of a single inlet and a single outlet through which circulates a volume V of fluid at a constant rate of flow F with T being the mean transit time of a particle. Its internal structure offers multiple pathways so that a particle of fluid entering into the system can then use different ways to reach the outlet with a variable dwell time inside the system. By assuming that the circulatory system is linear and time invariant (i.e., the probability function of the particle dwell time does not change with time, and the flow of indicator particles is representative of the observed flow), this theory enables the deduction of flow F, volume V, and mean transit time T from the local concentration-time curve C_*t*_(t) of the fluid at the outlet of the circulatory system. The principle consists of numerical deconvolution of the concentration-time curves to obtain the tissue response function and extract perfusion parameters. The most famous are the sparse singular value decomposition method (SVD) (Ostergaard et al., 1996) and Tikhonov regularization (Calamante et al., 2003). These methods are usually faster than model-dependent methods and are usually preferable for quantifying the MBF, although large differences between the results of these methods have been reported (Kudo et al., 2013). In addition, these techniques are sensitive to noise and contrast agent (CA) arrival delay to the myocardium (Jerosch-Herold et al., 2004b). Efforts have been made to increase the robustness of these techniques, as proposed by Jerosch-Herold et al. (2002), by combining SVD with Tikhonov regularization and B-splines. More recently, Wu et al. (2003) proposed an SVD variant that automatically truncates the singular value matrix to enhance the robustness to noise and replaces the arterial input function (AIF) matrix with a block-circulant matrix to make the method less sensitive to bolus arrival delay in the cerebral context.

None of these methods, SVD-based and model-independent methods, achieved sufficient robustness in clinics. The Fermi function is now considered the most popular and fully validated constrained deconvolution method (Jerosch-Herold et al., 1998; Pack and DiBella, 2010).

In parametric-indicator kinetic models, advanced methods proposed a Bayesian layer that intervenes within an added step of spatial regularization while modeling the residual function to estimate the kinetic parameters (Orton et al., 2007; Kelm et al., 2009; Schmid et al., 2009; Dikaios et al., 2017; Mittermeier et al., 2019). Note that Lehnert et al. (2018) proposed the spatial regularization of Tikhonov to try to also address this issue. In brief, all the uncertainties and issues resulting from a complex model involving recirculation, AIF dispersion during transportation from the estimated location to the point of measurement, and partial-volume and saturation effects have made the accurate estimation of hemodynamic parameters challenging, thus impeding the identification of true, reliable and useful quantitative myocardial perfusion markers for routine clinical use.

Recently, an original Bayesian approach has been shown to overcome most of the issues pertaining to the different estimation methods in brain perfusion (Boutelier et al., 2012; Kudo et al., 2014) promoting the use of simplified models to achieve robust pixelwise estimation. Our goal was to optimize and test this promising alternative approach (which innovatively proposes temporal regulation of the residuals function through prioritization) to ensure a robust estimation of MBF under the standard perfusion model. The first objective of this study was to compare the *in silico* performance of the Bayesian approach for myocardial tissue perfusion estimation with that of the most published variants of the Fermi function as a reference, and most often “clinically” used (despite a long processing time due to the number of processed convolutions needed to converge toward an acceptable solution). For this purpose, we designed a digital phantom composed of concentration-time curves generated with the 2-compartment exchange model and variable realistic ground truth perfusion parameters. The accuracy of the Bayesian and Fermi approaches was then assessed against this digital phantom. The second objective was to compare the Bayesian and Fermi MBF estimates in routine clinical settings in a pilot CAD population.

## Materials and Methods

### Study Population and CMR Acquisitions

**FIGURE 1:**
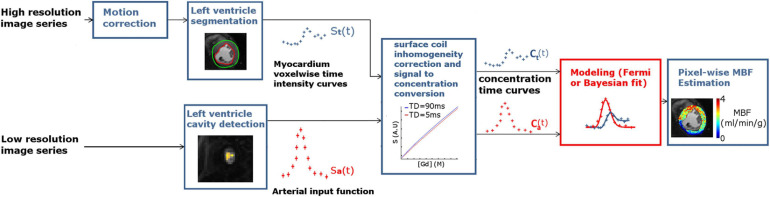
MBF measurement pipeline. Long-recovery-time, high-resolution image series (upper left) was acquired in basal, mid-ventricular, and apical locations at each timepoint (lower left); short-recovery-time, low-resolution images were also acquired to determine the AIF. High-resolution images are motion corrected to enable extraction of voxel-wise myocardium time-intensity curves. The AIF is extracted from the low-resolution image series by selecting the voxels inside the LV cavity and taking the maximum peak value. Time-intensity curves are then converted to time-concentration curves to avoid signal distortion before being given as input to the Bayesian and Fermi approaches.

Seventeen patients referred for known or suspected CAD with chest pain were examined with perfusion CMR. As explained below in detail, clinical data were used both to ensure the realistic calibration of *in silico* simulations and for the assessment in clinical settings. The study was performed with the approval of the institutional research ethics committee, and written informed consent was obtained from all the subjects. Patients were excluded if they had metallic implants or implanted cardiac devices incompatible with CMR, a glomerular filtration rate ≤ 30 ml/min, a high degree of atrioventricular block, severe chronic obstructive pulmonary disease or claustrophobia. Patients were asked to abstain from caffeine-containing products for ≥12 h prior to the CMR examination. The complete pipeline is described in the following section and is illustrated on Figure 1.

Perfusion imaging was acquired using a prototype dual-acquisition sequence as described by Gatehouse et al. (2004) on a 3T MAGNETOM Prisma (Siemens Healthineers, Erlangen, Germany) using an 18-element surface coil. A standard protocol was used with cardiac localization, steady-state free precession cine images acquired to cover the heart from the base to the apex. Vasodilation was obtained with a 0.4 ml bolus injection of regadenoson (Rapiscan, GEMS, Torrance, CA, United States) 1 min before imaging. Perfusion acquisition was then performed at 3 to 5 short-axis locations for every heartbeat with a bolus injection (6 ml/s) of gadoterate meglumine (0.2 mmol/kg) (Dotarem, Guerbet, Paris, France). The sequence acquired the first 3 proton density weighted scans planned for signal spatial normalization (flip angle of 5°) before imaging 3 to 5 T1-weighted frames. T1 weighting was obtained with a non-selective saturation recovery pulse train followed by saturation recovery time, defined as the duration between the end of the saturation pulse and the beginning of k-space acquisition. The acquisition kernel was a 2D single-shot turbo-fast low angle shot (FLASH) sequence. The main acquisition parameters of the high-resolution images were as follows: spatial resolution = 1.98 × 1.98 mm^2^, flip angle α = 10°, repetition time (TR) = 2 ms, echo time (TE) = 0.95 ms, long saturation recovery time = 43 ms, temporal parallel acquisition mode using generalized autocalibrating partially parallel acquisitions with an acceleration factor = 3, and linear k-space reordering. The main MR parameters of the AIF images were as follows: spatial resolution 5.94 × 5.94 mm^2^, flip angle α = 8°, TR = 1.3 ms, TE = 0.74 ms, a short saturation recovery time = 5 ms, and centric k-space reordering. For both acquisitions, the slice thickness = 8 mm, and the field of view = 380 × 380 mm^2^.

### Perfusion Image Preprocessing

Twelve patients were included among the initial 17 patients [5 were excluded due to electrocardiogram (ECG) and acquisition synchronization issues]. Perfusion-weighted images were registered offline using the Siemens non-rigid cardiac motion correction (MOCO) algorithm (Cerqueira et al., 2002) embedded in our custom MATLAB^©^ (R2013b, MathWorks, Natick, MA, United States) postprocessing pipeline. For the high-resolution perfusion-weighted images, surface coil inhomogeneity correction and conversion from signal time-intensity curves, S_*t*_(t), to concentration-time curves, C_*t*_(t), were performed using the approach proposed by Cernicanu et al. (Cernicanu and Axel, 2006). This method demonstrates the possibility of calibrating the signal within the perfusion-weighted image acquisitions by normalizing it with the proton density weighted images acquired at the beginning of the scan. Then, local CA concentration-time curves, C_*t*_(t), are deduced from the myocardial time-intensity curves, S_*t*_(t), that were extracted from the long inversion time acquisition (Gatehouse et al., 2004) with the use of look-up tables generated according to previously reported methodology (Cernicanu and Axel, 2006) and acquisition parameters. For each patient, the hematocrit value was quantified on site at the time of the CMR scan, and we assumed that tissue and large vessel hematocrit were equal.

The AIF time intensity curve, S_*a*_(t), was automatically extracted from the low inversion time acquisitions. Voxels considered for the AIF were identified according to the following criteria: being located inside the left ventricular (LV) cavity and having a peak signal value greater than 80% of the maximum voxel signal peak value. Voxels with signals trapped in the papillary muscle were excluded as proposed by Jacobs et al. (2016).

S_*a*_(t) was converted to an AIF time concentration function, C_*a*_(t), similarly to the myocardial C_*t*_(t) except that the longitudinal relaxation rate R_1_ was calculated according to the relationship given by eq. (1) (Kunze et al., 2017):

(1) $$S_{N}\,\left(R_{1}\right)=S_{0}\,\left(1-e^{-T\,D\cdot R_{1}}\right)$$

where S_*N*_ represents the signal acquired after N phase-encoding steps, that is, the k-space center of the image, which determines the overall contrast of the image. S_0_ is assumed to be equal to the proton density weighted signal obtained from the mean of the initial proton density scans, and TD is the delay time, i.e., the time between the end of the saturation pulse and the beginning of the k-space acquisition.

### Perfusion Quantification

Perfusion indexes were then estimated with the newly proposed approach and the reference approach, described in the two following sections.

#### Bayesian Approach

Bayesian deconvolution, described by Boutelier et al. (2012), was originally developed for cerebral blood flow estimation and is model independent (i.e., no strong assumption is made on the analytical form of the residue function). In this approach, the deconvolution is regularized by setting some prior information on the residue function: (i) it must be smooth and (ii) equal to 1 when the CA enters the voxel. The latter is naturally transferred into a Dirac probability for the prior on R(t = τ_*d*_): P(R(t = τ_*d*_))∝δ (R(τ_*d*_)−1), where τ_*d*_ is the arterial delay. The first prior information can be expressed as a condition on the second derivative that must remain small. It can be expressed as a prior probability with a Gaussian prior on the residue function R(t >τ_*d*_), with a mean of 0 and a covariance matrix corresponding to the second derivative operator. The smoothness prior is weighted by a new hyperparameter called ε that controls the strength of the smoothness and that needs to be estimated similar to any other parameter. Non-informative priors are used for the other parameters of the model. The posterior probability is computed by combining prior probabilities and the likelihood of the model by following Bayes’ rule. Each parameter of the model can be computed as the mean of the posterior, e.g., for MBF:

(2) $$M\,B\,F_{e\,s\,t}=\int M\,B\,F\times p\,\left(\theta \right)\,d\,\theta$$

where θ is a vector of [MBF, ε, τ_*d*_,R(t), σ] which are the parameters of the model, and p(θ), its probability. Boutelier et al. (2012) showed that the integral over R(t) and the noise σ can be calculated analytically, so the posterior reduces to a probability distribution function that depends on only three parameters: BF, θ, and ε. Then, the remaining integrals on BF, θ, and ε are computed numerically in a deterministic manner by sampling the posterior in the 3D parameter space (BF, θ, and ε) on a realistic range of values. Once BF, θ, and ε have been estimated, Boutelier et al. (2012) showed that the residue function can be estimated analytically by knowing those parameters. The mean transit time is computed as the area under the estimated residue function, and the myocardial blood volume is computed using the central volume theorem. Since the method is designed to solve the standard perfusion model, no assumption is made about the organ where it is applied, as long as the perfusion can be described as a convolution between a delayed AIF and a residue function. Hence, the same implementation can be used for brain or cardiac applications.

#### Fermi Function

Fermi modeling was introduced by Axel (1983) and has been widely used in myocardial perfusion analyses. This technique has been described in numerous studies, and its mathematical description has several variants according to the authors (Jerosch-Herold et al., 1998; Hsu et al., 2006, 2012; Jerosch-Herold, 2010; Broadbent et al., 2013). We chose to include in our study the mathematical description used by M. Jerosh-Herold (Fermi) without (Jerosch-Herold, 2010) and with management of bolus arrival time (BAT) delay (Jerosch-Herold et al., 1998) (called Fermi-δ in the following). Their descriptions are respectively, given by eqs. (3, 4).

Equation of Fermi residue function (without consideration of the BAT delay):

(3) $$r\,\left(t\right)=\frac{A}{1+e^{\left(t-\tau \,0\right)\times k}}$$

Equation of Fermi-δ residue function (with management of the BAT delay):

(4) $$r\,\left(t\right)=\frac{A}{1+e^{\left(t-\tau \,0-\tau \,d\right)\times k}}\times \prod \left(t-\tau_{d}\right)$$

where ∏ represents the unit step function, which equals 0 for τ_*d*_ < t and 1 for τ_*d*_ > t. By optimizing the parameters A, k, τ_0_, and τ_*d*_ and by convolving the resulting r(t) function with C_*a*_(t), one can obtain a fit of the concentration-time curve C_*t*_(t). The perfusion index MBF can then be estimated as the maximum amplitude of r(t) contributing to the observed curve C_*t*_(t). Attention should be paid to the convolution technique. A digital convolution with a large sample period, as in myocardial perfusion contrast enhanced MRI, can lead to numerical errors. For this reason, the AIF and residue time curves r(t) were linearly interpolated to reduce the sampling period by 20.

Only the first portion of the curve before the recirculation was considered to avoid increasing the bias in MBF estimation (Jerosch-Herold et al., 1998). The time window used to fit the curves was defined as the time between *t* = 0 s and the date where C_*a*_(t) reached its lowest value between the first-pass peak and the recirculation. Optimization was carried out by non-linear least squares fitting with the Levenberg-Marquardt algorithm, and the convolution operation was carried out in the Fourier domain to minimize the processing time. All processing was performed using MATLAB^©^ on a personal computer with an Intel (Santa Clara, CA, United States) Core i7 processor and 32 GB of RAM.

### Digital Phantom Synthesis

To evaluate the Bayesian approach and Fermi function, a numerical phantom dataset structure was created by generating tissue concentration-time curves C_*t*_(t) based on the approach proposed by Kudo et al. (2013), reflecting the true values for the MBF and the plasmatic volume values in myocardial diseases under stress and rest conditions. Synthetic datasets (performed at three different observation scale levels) were generated using a two compartment model (Jacquez, 1985; Sourbron and Buckley, 2012). This model assumes that the exchange is controlled in both directions by the permeability surface product P × S. The evolution of the CA concentration in both compartments is described by differential eqs. (5, 6):

(5) $$\frac{d\,C_{p}\,\left(t\right)}{d\,t}=F_{p}\,\frac{C_{a}\,\left(t\right)-C_{p}\,\left(t\right)}{V_{p}}+P\times S\cdot \frac{C_{i\,s\,f}\,\left(t\right)-C_{p}\,\left(t\right)}{V_{p}}$$

(6) $$\frac{d\,C_{i\,s\,f}\,\left(t\right)}{d\,t}=P\times S\cdot \frac{C_{p}\,\left(t\right)-C_{i\,s\,f}\,\left(t\right)}{V_{i\,s\,f}}$$

where, F_*p*_, V_*p*_ C_*p*_, and C_*isf*_ represent the plasma flow, plasma volume, plasma concentration, and interstitial fluid concentration, respectively.

The synthetic C_*t*_(t) curves were generated from input sets made with the following:

• A standard synthetic AIF designed as a combination of 2 Gaussian curves in accordance with features observed in patient datasets (baseline length, peak concentration, time to peak, and dispersion) and extracted from the true acquired Cardiovascular Magnetic Resonance Myocardial Perfusion datasets to match those observed clinically.
• A fixed value for the interstitial fluid volume (V_*isf*_ = 0.2 ml/g).
• A fixed value for the capillary permeability surface P × S constant (P × S = 0.95 ml/min/g) (Jerosch-Herold et al., 2004b).
• A combination of 10 plasmatic flow and 10 plasmatic volume values ranging from 0.48 ml/min/g to 3.9 ml/min/g (Gould, 2009) and from 0.04 ml/g to 0.1 ml/g, respectively, yielded a set of 100 combinations of plasmatic flows and plasmatic volumes.
• Delays of τ = 0 s, τ = 1.4 s, τ = 2.8 s, and τ = 3.5 s were chosen in the range of previously reported values in pigs (Jerosch-Herold et al., 2004a) or humans (Muehling et al., 2007). These delays were introduced by “shifting” the generated time curves on the time axis of an amount equal to the corresponding value of τ.

The C_*t*_(t) were generated over a time interval of 60 s, with a temporal resolution of 0.7 s as the average temporal resolution measured on clinical data. For numerical accuracy, all convolution operations described in this paper involved oversampling by linear interpolation.

The digital phantom structure is similar to that described by Kudo et al. (2013). It is composed of a 3D (F_*p*_, V_*p*_, time) array of tiles where each tile is a subarray component of size 10 × 10 × time in which the C_*t*_(t) curves are generated with identical perfusion parameters and added to centered Gaussian noise (Jerosch-Herold et al., 2002). The noise standard deviation was set based on the observations obtained from our clinical dataset at three representative scales:

• Global myocardium scale (for fast and global grading), where the measured Gaussian noise standard deviation is represented by the variable σ_*myo*_.
• Myocardial segment scale (American Heart Association segmentation), where the measured Gaussian noise standard deviation is represented by the variable σ_*segment*_.
• Voxel level, with the measured Gaussian noise standard deviation is represented by the variable σ_*voxel*_.

From our measurements, the standard deviations of the noise were σ_*myo*_ = 7.57 × 10^–6^, σ_*segment*_ = 7.83 × 10^–6^, and σ_*voxel*_ = 1.33 × 10^–5^.

**FIGURE 2:**
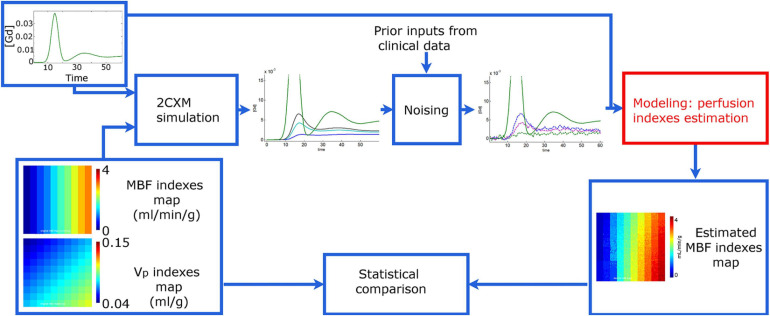
Simulation study principle. The synthetic time-concentration curves C_*t*_(t) are generated from a 2-compartment exchange model with arterial input function (AIF) C_*a*_(t) and maps of perfusion indices composed of 10×10 tiles. In any given tile, every model perfusion parameter is identical, and only the noise levels added to the generated curves C_*t*_(t) are different. Then, the myocardial blood flow (MBF) is estimated from each curve C_*t*_(t) with Fermi and Bayesian approaches. Finally, the output measurement maps are compared to those used for phantom generation. Phantom variants with variable noise and contrast-to-noise ratio but identical perfusion index maps and AIF were generated to assess the performance of both methods at myocardial-, segment- and voxel-level scales.

Therefore, three different phantoms were generated with the noise features matching each of the three observation scales. In this case, MBF was considered equal to the plasma flow F_*p*_ since no hematocrit was involved in the simulation. Figure 2 illustrates the entire process of digital phantom generation and MBF measurement.

### Data and Statistical Analysis

The data were screened for normality using the D’Agostino-Pearson omnibus K2 test (D’Agostino, 1986). Results were reported as medians and interquartile ranges, except where otherwise stated, when the normality assumption was not met.

#### Digital Phantom Data Analysis

**FIGURE 3:**
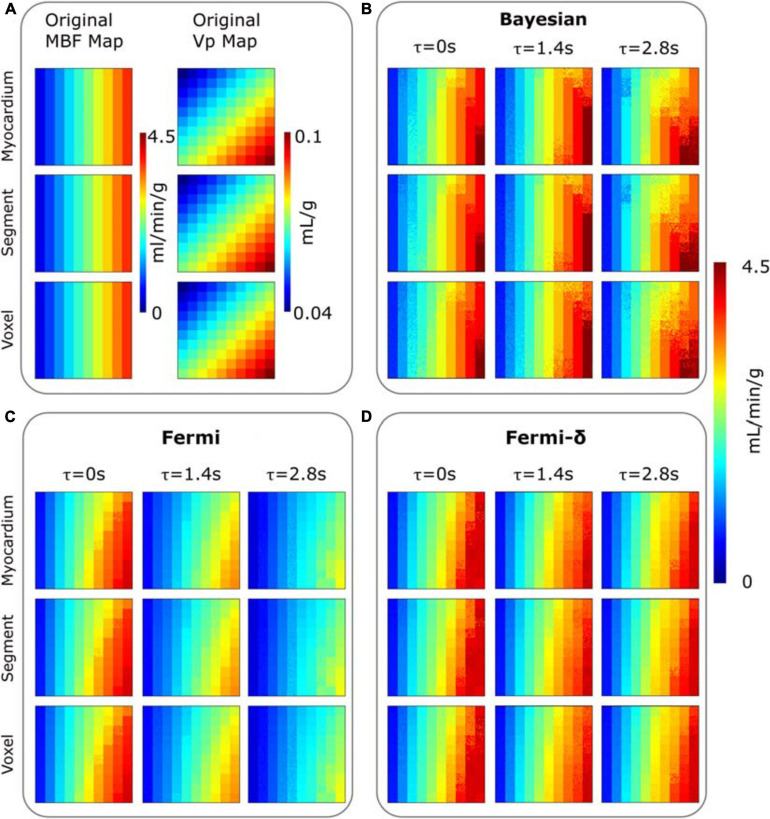
**(A)** Digital phantom of MBF (column 1) and plasmatic volume (V_*p*_) (column 2) maps composed of 10×10 tiles (delimited by grid squares). MBF maps estimated for three different delays (τ = 0 to 2.8 s) are provided with: **(B)** the Bayesian approach, **(C)** a Fermi function without delay management **(D)** a Fermi function including delay management (Fermi-δ. The different rows are corresponding to the clinical noise features added to the digital phantom time-concentration curves C_*t*_(t), and that correspond to the myocardium-, segment- and voxel-level observation scales, respectively. All the MBF maps (original and estimated) use the same color scale. A tile is composed of 10×10 curves C_*t*_(t) with identical perfusion parameters but different noise levels.

**FIGURE 4:**
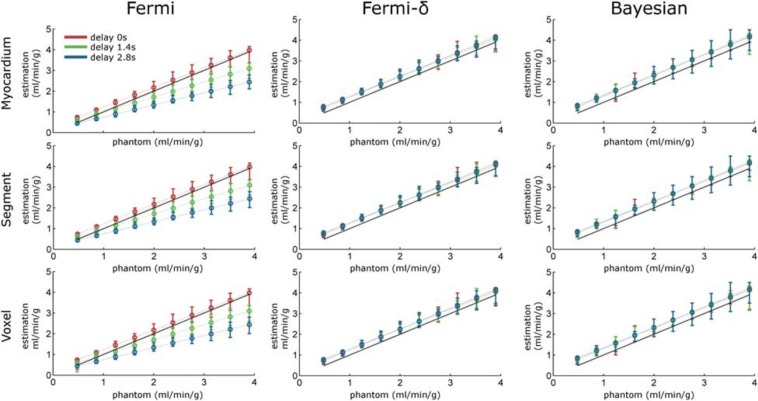
Regression line plots calculated over the MBF estimation maps with Fermi (without delay management, left column), Fermi-δ (with delay management, middle column) and Bayesian approaches (right column). The myocardium-, segment-, and voxel-level observation scales correspond to rows 1 to 3, respectively. The red, green, and blue dotted lines represent the regression lines for time delays of τ = 0 s, τ = 1.4 s, and τ = 2.8 s, respectively. The error bars indicate the maximum and minimum measurement values. The black dashed line is the identity function.

The agreement between the estimated MBF values calculated by the Bayesian, Fermi (eq. 2) and Fermi-δ (eq. 3) approaches was compared to the true reference MBF values that were also used as the input in the numeric phantom generation (Figure 3 shows the spatial representation, and Figure 4 shows the regression plots of the same data). The accuracy and precision of the estimates were quantified by Lin’s method. The Lin concordance correlation coefficient (Lin, 1989) is an accuracy measure and is the product of the Pearson correlation coefficient *r*, which measures how far each observation deviates from the best-fit line, a measure of precision, and C_*b*_, a bias correction factor that measures how far the best-fit line deviates from the identity function. The precision was also evaluated by calculating the relative error as the standard deviation, σ, of measurements of the 100 curves stored in each digital phantom tile divided by the target MBF value and by storing them into maps for visual analyses.

#### *In vivo* Data Analysis

Both processing approaches were then applied to the patient-level perfusion CMR datasets. For each patient, a pixelwise analysis was carried out on the basal, midcavity and apical slices after manual segmentation of the myocardium to reduce the processing time and allow the analysis to focus only on the LV wall voxels. After estimation, the MBF maps were then displayed as an overlay on the corresponding image series for visual analysis. The MBF measurement repartition is also displayed as a histogram. The histogram class amplitude was set to 0.2 ml/min/g. An expert analyzed the dynamic perfusion images and manually delineated normal and abnormal regions. From this analysis, we performed a statistical analysis on these two types of regions to compare the MBF measurements between the two areas.

Because reference values were not available in the *in vivo* dataset, the accuracy could not be evaluated. However, the precision was measured based on the wild bootstrap approach previously described in detail (Calamante and Connelly, 2009).

The principle of a wild bootstrap analysis is briefly described here:

• Calculate the residual *e*^∗^ vectors as the difference between C_*t*_(t) and Ĉ_*t*_(t), where Ĉ_*t*_(t) represents the fitted curve of C_*t*_(t).

(7) $$e^{*}=C_{t}\,\left(t\right)-\hat{C_{t}}\,\left(t\right)$$

• Modify the residual e^∗^ vectors to generate the wild bootstrap residuals.

(8) $$e_{i}^{\left(W\,B\right)}=a_{i}+\lambda_{i}+e^{*}$$

where *a*_*i*_ is the weighting factor included to produce the heteroscedasticity-consistent covariance matrix estimator and λ_*i*_ is the variable generated from a Rademacher distribution.

• A total of 1000 versions of wild bootstrap residuals are generated.
• Each version of e_*i*_^(WB)^ is added to the fitted Ĉ_*t*_(t).

(9) $$c_{i}^{\left(w\,b\right)}=\hat{C_{t}}\,\left(t\right)+e_{i}^{\left(W\,B\right)}$$

• The set of generated time curves, c_*i*_^(wb)^, are then processed by the tested approach. The average and standard deviation of MBF_*WB*_ are calculated.

The standard deviation of MBF_*WB*_ is considered to be the precision of the measurement.

For technical reasons of computational time, the wild bootstrap method was not used on all the voxels C_*t*_(t) but only on the most representative C_*t*_(t) of the normal and abnormal regions of each slice and patient. The most representative Ct, rep(t), was defined as follows:

(10) $$C_{t,r\,e\,p}\,\left(t\right)=\underset{C_{t}\,\left(t\right)\in R}{\arg\,\min}\sum_{t=0}^{T_{m\,a\,x}}{\left(\bar{C_{t,r}\,\left(t\right)}-C_{t}\,\left(t\right)\right)}^{2}$$

where R is the set of voxels’ C_*t*_(t) of a region of interest (ROI), T_*max*_ is the maximum acquisition time value, and C_*t,r*_(t) is the average region concentration-time curve.

Comparisons among the methods (Bayesian, Fermi and Fermi-δ) in normal and lesion regions were performed using the Friedman test with Dunn’s *post hoc* tests. Unpaired comparisons (normal vs. lesion regions) were obtained using the Mann-Whitney *U* test with the Hodges-Lehmann estimate of the difference between medians and 95% confidence intervals (95% CIs).

The statistical analysis was conducted using GraphPad Prism 7 (GraphPad, San Diego, United States) or Stata 15.1 (StataCorp, College Station, TX, United States). For all analyses, *p* < 0.05 indicated statistical significance.

## Results

### Performance of the Methods on Synthetic Datasets (Accuracy and Precision)

Figure 3 shows the MBF and plasmatic volume maps used for phantom generation and the MBF maps measured by both methods for the three different levels of noise, with and without delays of τ = 1.4 s and τ = 2.8 s (Supplementary Figure 1 provides the difference maps: known reference MBF – estimated MBF).

A visual analysis of maps is very useful for rapidly identifying the differences between the real and synthetic data and the differences between the approaches. When there is no delay, the estimation maps of all the methods show a vertical gradient. This effect is clearer for higher values of the MBF located on the right sides of the maps. This finding suggests that the MBF measurement is influenced by the plasmatic volume V_*p*_. Indeed, one can observe that for the same theoretical MBF values (phantom maps), the MBF estimates are smaller when V_*p*_ is smaller (top right corner) and increase as V_*p*_ increases (bottom right corner). This effect can be observed in the results of each method. However, the measurement remains fairly insensitive to the noise level.

The introduction of delays in C_*t*_(t) (τ = 1.4 s or 2.8 s) revealed clear differences among the methods. We observed a clear measurement underestimation with the Fermi function with no delay management, especially in the higher MBF values. This underestimation is related to the combination of the delay and the variation in V_*p*,_ as shown above. The use of the BAT delay parameter in the Fermi-δ function enabled us to handle this issue and gave estimation results comparable to those obtained without delay introduction. The Bayesian measurement also remained fairly stable. One can, however, observe a slight increase in the vertical gradient.

The quantitative analyses of the same data are provided in Figure 4 and Table 1. Without delay and under all noise conditions, the Bayesian estimates showed a regression slope (an estimate of the proportional differences with reference values) near the identity (equal to 0.98). The intercept (an estimate of the systematic differences) was close to 0.34, meaning there was a slight but constant overestimation of the MBF values. The Fermi function with no delay management showed a slope of 1.21 and systematic differences equal to 0.21. The Fermi-δ function yielded a slope of 1.01 and an intercept of 0.23. Hence, under these noise and delay conditions, all approaches show excellent fidelity to the ground truth parameters.

**TABLE 1:** Relationships among the Bayesian, Fermi, and Fermi-δ simulated myocardial blood flow (MBF) estimates (10 MBF × 10 V_*p*_ × 100 noise realizations) compared to the MBF reference values.

		Bayesian				Fermi				Fermi-δ			
		R^2^	Slope	Intercept	Proc. time (s)	R^2^	Slope	Intercept	Proc. time (s)	R^2^	Slope	Intercept	Proc. time (s)
Myocardium	τ = 0 s	0.98	0.983	0.339	9	0.98	1.121	0.205	724	0.98	1.011	0.229	317
	τ = 1.4 s	0.97	1.020	0.269	20	0.97	0.744	0.257	527	0.98	0.947	0.339	289
	τ = 2.8 s	0.91	0.962	0.395	22	0.96	0.630	0.168	830	0.97	0.972	0.315	280
Segment	τ = 0 s	0.98	0.983	0.338	9	0.99	1.121	0.205	491	0.98	1.012	0.229	314
	τ = 1.4 s	0.97	1.018	0.271	17	0.97	0.744	0.265	532	0.98	0.950	0.338	286
	τ = 2.8 s	0.91	0.961	0.397	20	0.96	0.629	0.169	796	0.98	0.972	0.315	315
Voxel	τ = 0 s	0.98	0.984	0.336	8	0.98	1.121	0.206	482	0.98	1.011	0.228	398
	τ = 1.4 s	0.97	1.015	0.283	19	0.97	0.744	0.257	502	0.98	0.950	0.334	301
	τ = 2.8 s	0.91	0.95	0.405	20	0.96	0.629	0.171	827	0.97	0.974	0.312	297

*The ordinary least squares (OLS) regression models were obtained for different time delays (τ varying from 0 to 2.8 s) and different noise levels (myocardium, segment, or voxel level). (*R*^2^, coefficient of correlation, slope and intercept of the OLS regression model, processing time in seconds; τ, simulated time delay).*

The introduction of delays (τ = 1.4 s and τ = 2.8 s) led the Bayesian estimates to a slight reduction in the correlation coefficient *r*^2^ (*r*^2^ = 0.97 and 0.91, respectively). Regarding the regression line, no noticeable changes were observed in the slope and intercept values. The Fermi function with no delay management estimates showed a marked decrease in the linear regression slopes, down to 0.74 and 0.63, respectively. This result indicates a proportional underestimation with increasing MBF values. This underestimation is clearly visible in the MBF maps in Figure 3 and in the regression line plots in Figure 4. In contrast, the Fermi-δ function with the management of delays remains stable, as the correlation remains almost constant under all noise and delay conditions. Delays lead to a small reduction in the slope of the regression line and an increase in the systematic error. Therefore, the introduction of delay management in the Fermi function leads to a better accuracy for the MBF measurements when the BATs are considered.

Table 2 shows Lin’s concordance correlation coefficient analysis and the minimal impact of various scales and delays on both the precision and accuracy of the Bayesian estimates of the numerical phantom data. While the Fermi-δ function shows a similar pattern, the Fermi function without delay management (middle) exhibits a clear loss of accuracy with increasing time delays since C_*b*_ is decreasing overall from 0.92 to 0.73.

**TABLE 2:** Agreement between the simulated Bayesian, Fermi, and Fermi-δ myocardial blood flow (MBF) values and the reference MBF values.

		Bayesian			Fermi			Fermi-δ		
		CCC	ρ	C_*b*_	CCC	ρ	C_*b*_	CCC	ρ	C_*b*_
Myocardium	τ = 0 s	0.953 (0.952–0.954)	0.990	0.963	0.913 (0.910−0.915)	0.994	0.918	0.963 (0.961−0.964)	0.988	0.974
	τ = 1.4 s	0.947 (0.946−0.949)	0.985	0.961	0.904 (0.902−0.906)	0.986	0.917	0.966 (0.964−0.967)	0.988	0.977
	τ = 2.8 s	0.916 (0.913−0.919)	0.954	0.961	0.718 (0.712−0.724)	0.984	0.729	0.961 (0.960−0.962)	0.987	0.973
Segment	τ = 0 s	0.953 (0.951−0.954)	0.990	0.963	0.913 (0.910−0.915)	0.994	0.918	0.962 (0.961−0.963)	0.988	0.974
	τ = 1.4 s	0.947 (0.946−0.949)	0.985	0.962	0.904 (0.902−0.906)	0.986	0.917	0.966 (0.964−0.967)	0.988	0.977
	τ = 2.8 s	0.916 (0.914−0.919)	0.954	0.961	0.718 (0.712−0.723)	0.984	0.729	0.961 (0.960−0.962)	0.987	0.973
Voxel	τ = 0 s	0.954 (0.952−0.955)	0.990	0.963	0.912 (0.910−0.915)	0.994	0.918	0.962 (0.961−0.963)	0.988	0.974
	τ = 1.4 s	0.946 (0.944−0.947)	0.985	0.960	0.904 (0.902−0.907)	0.986	0.917	0.966 (0.965−0.967)	0.988	0.977
	τ = 2.8 s	0.919 (0.916−0.921)	0.957	0.960	0.718 (0.712−0.723)	0.984	0.729	0.961 (0.960−0.962)	0.987	0.973

*The Lin concordance correlation coefficient (CCC) can be expressed as the product of the Pearson correlation coefficient ρ (a precision measure) and C_*b*_ (an accuracy measure).*

Table 3 summarizes the average and maximum relative error of the MBF index estimations of the curves stored in each tile generated with the identical perfusion index values. The average and maximum relative errors were lower with the Fermi model without delay management, with the average relative error ranging from 0.7 to 1.9% but with the maximum relative error ranging from 3.1 to 10.1%. The relative error slightly increased with increasing noise to reach a maximum relative error of 14.3%. The use of the Fermi-δ function results in an increase in the average relative error (from 1.5 up to 2.2%), but the maximum relative error was lower, ranging between 4.8 and 6.5%.

**TABLE 3:** Averages and maximum relative errors with Fermi modeling and the Bayesian approach.

		Bayesian		Fermi		Fermi-δ	
		Avg (%)	Max (%)	Avg (%)	Max (%)	Avg (%)	Max (%)
Myocardium	τ = 0 s	2.3	12.1	0.7	3.5	1.5	6.1
	τ = 1.4 s	2.9	11.1	0.9	5.5	1.3	4.8
	τ = 2.8 s	2.8	12.6	1.4	7.6	1.3	5.5
Segment	τ = 0 s	2.3	12.1	0.7	3.1	1.5	6.1
	τ = 1.4 s	2.9	10.8	0.9	5.5	1.3	5.5
	τ = 2.8 s	3.0	13.2	1.4	7.4	1.4	5.8
Voxel	τ = 0 s	3.7	12.6	1.4	10.0	2.2	6.5
	τ = 1.4 s	3.7	9.6	1	10.1	2.0	4.9
	τ = 2.8 s	4.2	11.7	1.9	7.7	1.9	5.5

*The measurement standard deviation was calculated based on the 100 curves stored in the digital phantom tiles.*

The Bayesian average and maximum relative errors ranged from 2.3 to 4.2% and from 9.6 to 13.2%, respectively. With this method, we observed a slight increase in the average relative error by introducing noise and delay, but the maximum relative errors remained stable.

These results against the simulated data show, among other points, the importance of BAT delay introduction in the Fermi function to the accuracy. In the following section, the study further focuses only on the Bayesian approach and the Fermi-δ function with delay management.

**FIGURE 5:**
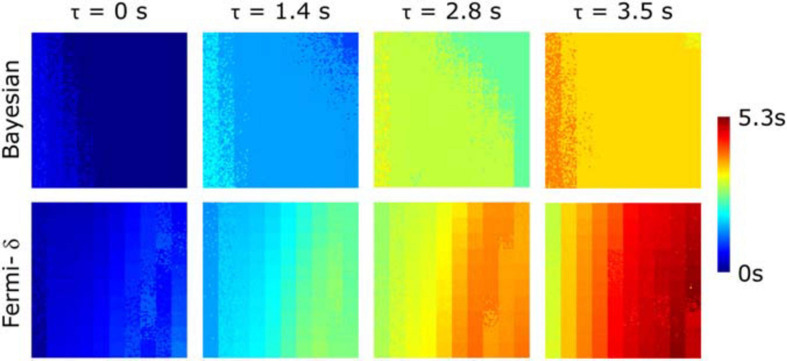
Estimated delay maps calculated with Bayesian and Fermi with delay management (Fermi-δ) methods for 4 predefined delays (τ = 0 s, τ = 1.4 s, τ = 2.8 s, τ = 3.5 s) on time-concentration curve noise measured at the voxel-level observation scale using the MBF and V_*p*_ pairs displayed in Figure 3. The Bayesian approach gives stable and accurate estimations for a large range of values with the exception of lower MBF values (left side of the maps) and for the higher MBF values combined with medium plasmatic volumes (right top corner). Fermi-δ shows a bias in its estimation of the delay as a function of MBF and plasma volume, tending to overestimate it.

Figure 5 shows the estimations of the delays given by the Fermi-δ and Bayesian approaches against the simulated data. For τ = 0 s, the Bayesian technique tends to slightly overestimate the delay for lower MBF values but remains very stable and accurate beyond a threshold value of 1 ml/min/g. The reason for this overestimation is the low enhancement of the concentration-time curves. Conversely, the Fermi-δ function showed a maximum overestimation for higher values of flow, decreasing with the MBF and V_*p*_. The influence of perfusion parameters is even more trivial in maps with delays of τ = 1.4 s and τ = 2.8 s. When delays are introduced (τ = 1.4 s and τ = 2.8 s), the Bayesian approach stays extremely stable except for the combinations of the MBF and V_*p*_ in the top right-hand corner, where the high MBF values are combined with moderate plasmatic volume values. In this area, the underestimation remains constant and lower than on the maps obtained with Fermi modeling.

### Comparison of the Fermi-δ and Bayesian Results and Precision on the Clinical Datasets

**FIGURE 6:**
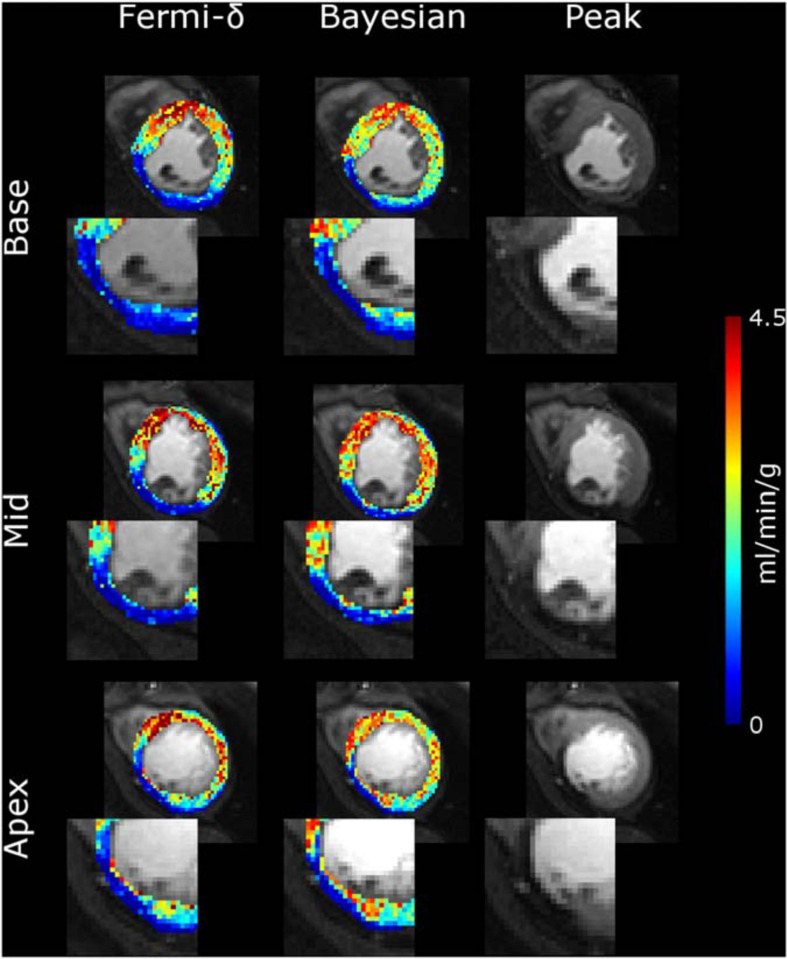
MBF maps calculated for a patient over the basal, mid-ventricular and apical slices with the Fermi-δ function (column 1) and a Bayesian framework (column 2). Column 3 shows the peak of the myocardium contrast enhancement image. All the MBF maps use the same color scale. The ischemia on the inferolateral segment of each slice is more clearly depicted with Bayesian modeling than with the Fermi-δ function because the former has a larger range of values.

Figure 6 shows the pixelwise MBF estimation with Fermi-δ modeling and the Bayesian approach for the basal, midcavity, and apical slices of a specific patient. This patient was referred for chronic inferoseptal inducible ischemia. One can observe a gradient from the center of the lesion to the normal region, suggesting a better capability of the Bayesian technique to differentiate fine MBF variations.

**FIGURE 7:**
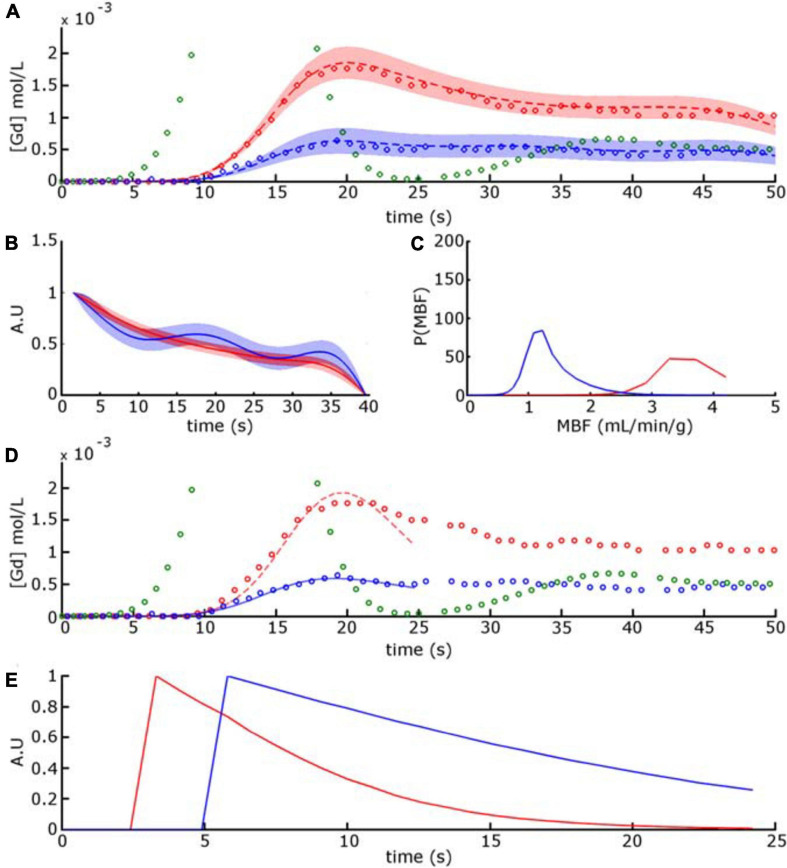
Red circle and blue circle [in **(A,D)**] corresponds to concentration-time curves of voxels selected in normal and abnormal regions from the mid-ventricular image series displayed in Figure 6. The green circle time curve represents the AIF C_*a*_(t), truncated for display purposes. In **(A)** the dashed red and blue curves are the time-intensity curves obtained with the Bayesian approach. The fill plots, around the dashed curves and residue functions, indicate the confidence intervals within which the temporal values of functions were estimated. **(B)** Unitary residue functions and **(C)** MBF posterior probability functions calculated for the Bayesian approach. In **(D)** the red and blue plain curves are the time-intensity curves obtained with the Fermi-δ method. **(E)** Unitary residue functions calculated for the Fermi-δ function.

Figure 7A shows the C_*t*_(t) curves selected from the normal and abnormal regions in Figure 6 and their fitted values with the Bayesian technique. The fill plots around the fit and residue functions indicate the CIs within which the temporal values of functions were estimated. Unitary residue function behaviors (Figure 7B) indicate a biexponential decrease with a steeper slope in the first part of the time interval that flattens in the second part before decreasing to 0. The latter behavior occurs because the residue function was assumed to reach zero after a maximum time elapse of *t* = 40 s. This value was empirically defined by the optimization of a digital phantom dataset. One can also notice delays occurring between C_*a*_(t) and C_*t*_(t) in both regions. The delays were measured as the time elapsed between the moment when C_*a*_(t) reached 10% of the first-pass peak value and the moment when C_*t*_(t) reached 10% of its own peak value. In this example, delays in the normal and abnormal regions were found to be τ_*normal*_ = 2.8 s and τ _abnormal_ = 5.4 s.

Figure 7B shows C_*t*_(t) and the fitted and unitary residue functions selected from the previous voxel output by the Fermi-δ method. The residue functions were normalized for the sake of visualization. In both the normal and abnormal regions, the shape of the residue function describes a monotonic decay at a near-exponential rate and disables this approach to accurately describe exchanges between plasmatic and extracellular and extravascular spaces. It also displays the respective MBF probability distribution for these functions, where the mean corresponds to the estimated MBF value.

For all patients and in the normal regions, there were significant differences among the methods [χ^2^(3) = 16.24, *p* < 0.001]. While the normal Bayesian MBF median values were close but slightly higher than the Fermi-δ MBF estimates [2.59 (2.20, 3.60) ml/min/g vs. 2.53 (1.95, 3.27) ml/min/g, respectively, *p* = 0.65], the Fermi MBF estimates were significantly lower [1.94 (1.54, 2.87) ml/min/g] than both the Fermi-δ (*p* = 0.02) and Bayesian (*p* = 0.0002) MBF estimates. In abnormal regions, we observed significant differences among the methods [χ^2^(3) = 24.63, *p* < 0.001]. The Bayesian MBF median values [1.19 (0.51, 1.9) ml/min/g] were significantly higher than both the Fermi-δ [0.64 (0.39, 1.02)] and Fermi MBF values [0.60 (0.47, 0.88)] (both *p* < 0.001). The methods showed significant differences between the normal and abnormal regions (all *p* < 0.0001).

**FIGURE 8:**
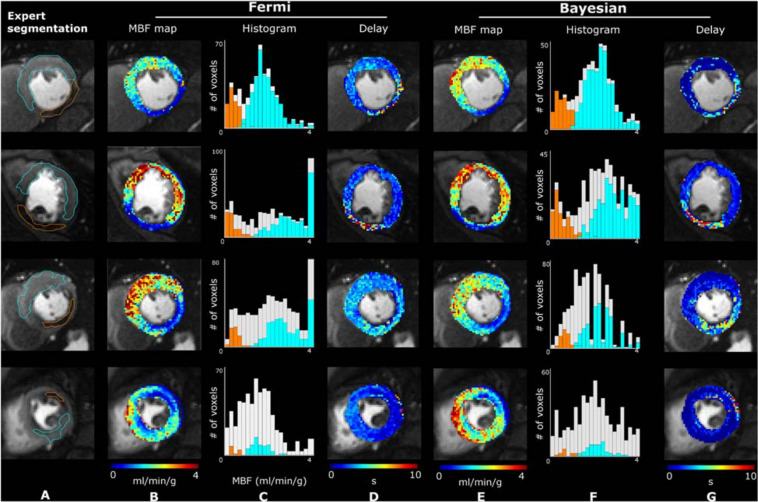
Illustrative peak contrast images, MBF and delay maps obtained on four different patients. Column **(A)** reports expert segmentation of the normal (cyan contour) and abnormal (orange contour) regions. The right side of the figure sequentially displays the estimated MBF maps with the Fermi function with delay management [column **(B)**] and the Bayesian method [column **(E)**] and their respective histograms [columns **(C,F)**], highlighting bins belonging to the segmented myocardium (light gray) and to normal (cyan) and abnormal (orange) regions. The delay maps [columns **(D,G)**] were calculated from the Fermi and Bayesian approaches, respectively.

Figure 8 displays the MBF maps (in ml/min/g) and the delay maps obtained with the Fermi-δ and Bayesian methods. These maps are displayed over the peak image of the midcavity slice from four different patients, with normal (cyan) and abnormal (orange) regions drawn by a medical expert. The MBF map values are also shown as a histogram, and the values corresponding to normal and abnormal regions are highlighted with their respective colors over the global myocardium histogram (gray) to facilitate the visualization of the region repartitions. The maps provide good information on the spatial repartition of the MBF values over the LV myocardium, enabling the appropriate localization of an abnormal perfusion defect. The histogram provides additional information on the repartition of MBF values in both regions, highlighting the visual difference between regions while offering an appreciation of the value of the hyperemic MBF and its variability among subjects. More precision on Bayesian delays can be found in Supplementary Figure 2.

**FIGURE 9:**
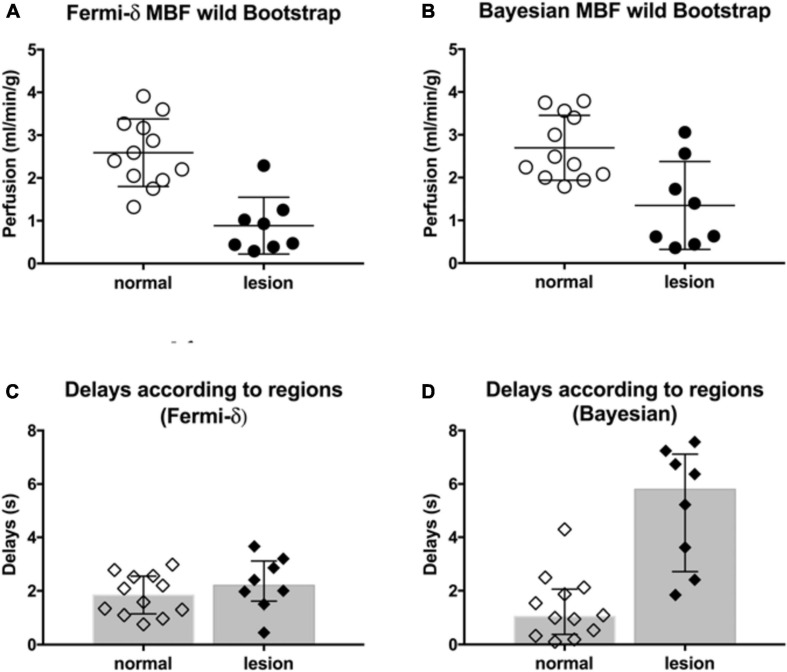
(Top) Average MBF values (ml/min/g) obtained by wild bootstrapping with the **(A)** Fermi-δ and **(B)** Bayesian methods in normal (empty circles) and abnormal (black-filled circles) regions (i.e., ischemic lesions, if any) from all subjects enrolled. The MBF estimation was carried out on the most representative single pixel C_*t*_(t) out of all the ROIs and in all patients, which was defined by medical experts. (Bottom) The delays measured in the normal (empty diamonds) and abnormal (black-filled diamonds) regions with the Fermi-δ and Bayesian approaches are reported in plots **(C,D)**.

Figure 9 plots the mean MBF values (in ml/min/g) obtained with the (A) Fermi-δ and (B) Bayesian methods in normal and abnormal regions for all the enrolled subjects obtained by a wild bootstrap (WB) analysis carried out on the most representative single pixel C_*t*_(t) of the ROIs defined by a medical expert. The Bayesian estimates of the MBF in not only normal but also abnormal regions were globally higher than the MBF estimated with the Fermi-δ method. In ischemic regions, the difference among the methods was more pronounced and coherent with simulations that showed an underestimation of the Fermi-δ algorithm in the case of delays. As expected, by using the Bayesian approach, there was a marked difference in the BAT between the normal and abnormal regions with a median delay in the normal regions of 1.04 s [0.61, 2.14] and in abnormal/hypoperfused regions of 5.79 s [3.25, 6.99] (Figure 9D). The median difference was 4.23 s [1.6, 5.8], with *p* < 0.001. The Fermi-δ approach, on the other hand, failed to extract differences in the BAT between the normal regions and the hypoperfused regions (*p* = 0.38), with delays of 1.83 s [1.36, 2.33] and 2.20 s [1.41, 3.1], respectively (Figure 9C).

## Discussion

This work introduces a new method to obtain robust cardiac perfusion indexes and has the ability to clarify the available Fermi variants’ capability and/or bias. Despite being considered the gold standard in previous clinical studies, Fermi models exhibit considerable variations in implementation and mathematical description (Jerosch-Herold et al., 1998; Hsu et al., 2006, 2012; Jerosch-Herold, 2010; Broadbent et al., 2013). As cited in the introduction, the use of Bayesian approaches and prior information for multiple kinetic parameter estimation in dynamic contrast enhancement with complex modeling has been explored before (Orton et al., 2007; Kelm et al., 2009; Schmid et al., 2009; Dikaios et al., 2017; Mittermeier et al., 2019). However, the complexity of the models relative to the cardiac data, characterized by reduced sample time due to breath-hold duration and more variability related to motion, led to difficulties in model fitting with multiple distinct combinations of parameters estimation low precision at the clinically observed noise level further failing to convince clinicians. Even using the simplest Fermi model, fitting was reported to fail in 10% of cases using concentration curves averaged over a segment of the myocardium (Broadbent et al., 2013). Lehnert et al. (2018) recently proposed an approach relying on Bayesian inference combined with Tikhonov regularization. If the results were encouraging, the methodology was tested on a very small number of subjects (5 patients with angina symptoms) and the study did not investigate the sensitivity to BAT. The same team (Scannell et al., 2020) also confirmed very recently in 8 patients that an approach relying on hierarchical Bayesian modeling was of interest for multi-parametric estimation. Their work similarly cares to beforehand validate the methodology on a digital phantom, constructed with synthetic myocardial time concentration curves generated from 2-compartment exchange model. Their results show high accuracy on the simulated data, with values comparable to ours for myocardial blood flow in the ischemic and remote lesions in the few analyzed clinical datasets. Our study proposes extended simulation results with a wider range of flow and volume values integrated to our digital phantom, and reinforce the clinical results. Indeed we investigated the ability of Bayesian approach to address the challenge of accurately assessing myocardial hemodynamic parameters in a larger number of patients with ischemic lesions. We show that the implemented Bayesian approach does not perform better than the Fermi methods in our *in silico* study for MBF estimation, but is the only one to jointly estimate delays accurately. Moreover, in the clinical study, we clearly illustrate that it offers much more convincing results *in vivo* with a clear superiority to reveal differences in the MBF and delays in myocardial tissue, which is of crucial interest in the explored cohort of patients.

First, in this work, using *in silico* modeling of myocardial perfusion regimens to account for perfusion delays and a broad range of clinically realistic conditions, we assessed the reliability of the MBF estimates using the Bayesian approach. The main finding, from a clinical perspective, was that the MBF estimates provided by the Bayesian approach are remarkably stable throughout the whole range of perfusion regimens and even at high MBF values with a systematic bias of less than 0.5 ml/min/g while remaining insensitive to delays in CA arrival at the myocardium. We also confirm that the Fermi function without introduction of a delay variable is very sensitive to perfusion delays and, under these conditions, suffers from proportionally biased measures with a severe MBF underestimation. The introduction of a BAT delay parameter τ_*d*_ is necessary to yield accurate MBF measurements although rarely clearly mentioned in the literature (Cheong et al., 2003; Zarinabad et al., 2014). From a clinical translation perspective, the *in vivo* results obtained in a small exploratory cohort clarify these *in silico* findings and show how a comparison between the Bayesian and Fermi-δ estimates within normal and abnormal regions in CAD subjects can turn into differences with a true clinical impact. Of interest is the significance of the CA arrival time delays (with a median value of 5.8 s in abnormal regions up to a maximum of 7.6 s, see Figure 9) and the real importance of introducing a delay in Fermi modeling without which the MBF measurements are strongly underestimated. CA arrival time delays remain insufficiently considered in cardiac perfusion analysis workflows and have been identified as useful markers of collaterality that also help predict the flow capacity (Jerosch-Herold et al., 2004a; Muehling et al., 2007). Delay maps not only contain intrinsic pathophysiological information on vascular bed function but also may be able to serve as quality control in the clinical use of Fermi perfusion maps to warn of delays that cannot be dismissed. The similar trends in the results obtained when applying MBF calculations using different approaches are summarized in Figure 9 in both normal and abnormal ROIs; these trends are very reassuring regarding the stability of the postprocessing at all noise levels, demonstrating an independence with regard to the signal-to-noise ratio (SNR)/contrast-to-noise ratio (CNR) of the estimation using a simple model.

In the current study, we objectively compared the accuracy and reliability of the two linear shift-invariant approaches (Fermi-δ vs. Bayesian algorithms) by assessing their performance on an especially designed digital phantom based on the widely accepted indicator kinetic theory. We implemented real condition noise levels in the myocardium that we extracted from clinical datasets in which the true values of the MBF, the plasmatic volume (V_*p*_), the capillary permeability–surface area product (P × S), and the indicator arrival delay (τ) are known variables, with perfusion parameter ranges based on well-established cardiovascular imaging literature (Jerosch-Herold et al., 1998; Gould, 2009). The comparisons between the theoretical and estimated maps allow one to rapidly visualize the errors in the MBF and the differences in their distribution as a function of coupled MBF and V_*p*_ pairs prior to generating more detailed regression line plots and performing correlation analyses.

Figures 3, 4 reveal that the Bayesian approach tends to overestimate the MBF index and shows the sensitivity of the Fermi approach to delay CA arrival when the latter is not considered. The regression line coefficients also calculated on regression line plots provide good information on the measured linearity against the range of MBF values. These results agree with previous observations but highlight the dramatic loss in estimated linearity by the Fermi model when a time delay is introduced and not considered in the model. In contrast, the Bayesian method remained very stable with time delay introduction.

Fermi residue function decay is based on a single exponential parametric function with an initial “shoulder” that addresses the period during which the CA has not yet left the compartment. However, the tissue’s physiological behavior is a fast decay during a first-pass representative portion of the plasmatic transit followed by a slower decay, describing the dwelling of a portion of the CA in the extracellular and extravascular space. We reduced the C_*a*_(t) and C_*t*_(t) time interval analysis from the beginning of the baseline to the end point set as the minimum CA concentration between the first and second pass in the blood pool to compensate for this phenomenon (Jerosch-Herold, 2010). In the case of a slowly rising curve, the peak value can be reached after this time interval, leading to a dramatic underestimation of the MBF. The Bayesian approach is not exposed to this problem since the residue function shape is not constrained by an analytical expression. Hence, as described in Figure 7, the Bayesian residue function provides a more realistic description of physiological tissue behaviors and could even provide information on the surface product permeability and extracellular extravascular space.

Figures 3, 4 also highlight the plasmatic volume influence on the MBF measurements for all methods as a vertical gradient emphasized on the MBF estimation maps in the bottom corner of the figures. This observation agrees with the observations of Kudo et al., in a similar study (Kudo et al., 2013). We also demonstrated that the Bayesian and Fermi-δ methods were robust to noise, as attested by the correlation coefficient *r*^2^ (0.97 ≤ *r*^2^ ≤ 0.98 with the Fermi function, and 0.91 ≤ *r*^2^ ≤ 0.98 with the Bayesian technique). In a similar study, Kudo (Kudo et al., 2013) considers that a good correlation is reached when the Pearson’s correlation coefficient *r*^2^ reaches values greater than 0.9. This noise insensitivity may be due to the injected CA dose of 0.2 mmol/kg, as a lower dose (Jerosch-Herold et al., 1998; Kellman et al., 2017; Kunze et al., 2017) is usually injected in similar studies.

Figure 7A also shows the oscillation in the residue function for the C_*t*_(t) curve associated with voxels in abnormal regions. The Bayesian algorithm is a non-parametric deconvolution approach, for which the monotonicity and positivity constraints are not implemented. In contrast, the Fermi model uses an analytical function and naturally “embeds” such constraints through the choice of the model. The oscillating or negative residue function can be returned by the Bayesian algorithm. This phenomenon is a defect of most non-parametric deconvolution algorithms, such as the well-known SVD family of algorithms. This effect is more likely to appear when noise or some artifacts affect the concentration-time curve. A careful observation of the whole time-intensity curves systematically reveals slight oscillations after a BAT delay, probably due to limitations resulting from the MOCO algorithms, which are impossible to simulate and are not considered here (these oscillations introduce “noise” that may not be considered Gaussian but are unaddressed intrinsically by our simulations). These limitations most likely explain the oscillations in the residue function estimated by the Bayesian technique and are most apparent at a low SNR, as encountered in the hypoenhanced region of the ischemic myocardium.

The Bayesian approach provided further worthwhile information such as the probability density function of the MBF measurements and the temporal uncertainty of both the fitted and residue function, as shown in Figure 7A. This information allows a simple evaluation of the measurement reliability and is particularly useful at the voxel-level observation scale, where the concentration-time curves may be contaminated by errors introduced by image calibration and registrations. However, this information is provided only by the Bayesian method.

The *in vivo* results confirmed the *in silico* simulations, as previously discussed and as illustrated in Figure 9. The results corroborate the hypothesis that the delays are likely to explain the differences observed in the clinical data between the two algorithms. Note that in our sample of patients referred for CAD detection, our results suggested heterogeneity in the MBF maps that was related to variable delays among and within regions, as shown in Figure 8, which is information that is typically not accessible in clinical scenarios with the visual inspection of images at peak intensity. In practice, and from a clinical perspective, the influence of CA arrival time delays with the Fermi-δ approach requires delay management to avoid the proportional underestimation of the MBF with increasing flow and delays, which means potentially misleading results for midrange to high-range MBF levels and therefore an overestimation of disease severity for a constant delay across regions. It also suggests that MBF together with delay maps are likely valuable tools for accurate MBF estimation in ischemic patients referred for CAD detection. Indeed, in this patient population, perfusion will more strongly depend on collateralized segments, especially in patients with chronic total occlusion but also after myocardial infarction. The effects of P × S and V_*isf*_ were not studied in this paper because of the volume of data required but would be of great interest in future work.

Our MBF estimates are in the range of results reported in recent CMR studies in CAD patients. Using Fermi models, Kotecha et al. (2019) reported stress perfusion values of 1.47 ± 0.48 ml/g/min, while Hsu et al. (2018) reported lower values of 0.92 ± 0.36 ml/g/min. In remote normal regions, Kotecha et al. (2019) reported stress values of 2.47 ± 0.5 ml/g/min, while Hsu et al., measured stress values of 2.32 ± 0.57 ml/g/min. Scannell et al. (2020) using a Bayesian model, reported stress perfusion median values of 2.35 (1.9, 2.68) ml/g/min, while in lesions, values were below 1.0 ml/g/min according to the provided color scale in the provided graphs. Similar ranges of stress and rest values were also found by Kellman et al. (2017); Knott et al. (2019), and Brown et al. (2018), who used all the same methodology and software (Kellman et al., 2017). The synthetic AIF concentration-time curve C_*a*_(t) used for dataset generation was designed from observations made on a set of patients. Only a few voxels were selected for AIF extraction, considering that the rest of the LV blood pool was contaminated by partial-volume effects. Note that the volume from low-resolution image voxels is equivalent to that of approximately 4-fold voxels from the high-resolution images, giving a reasonably high CNR for the measurement of features (baseline length, time to peak, and maximum peak value). The average CA concentration at the peak value was 5.86 mmol/L at stress, which was slightly more substantial than that observed by Kellman et al. (2017) but was consistent, since a dose of only 0.05 mmol/kg was injected at a rate of 6 mL/s in this study instead of a full dose of 0.2 mmol/kg injected at a rate of 4 mL/s. In their study, which used a 3T scanner similar to ours, Papanastasiou et al. (2016) measured AIF peak values ranging from 5.5 mmol/L to 6.5 mmol/L with identical injection parameters as was done in Kellman’s study. The noise parameters were measured at the base of the LV myocardium at the different observation scale levels method, with slight, rather constant and thus fairly acceptable theoretical bias.

In conclusion, the Bayesian method can be considered an appropriate model-independent method to be used when myocardial perfusion quantification is needed. Our mixed *in silico* evaluation coupled with a preliminary clinical study shows the efficiency of the method, relying on its independence of CA delays, and its ability to distinguish normal from abnormal myocardial regions while being a fast, robust, and unbiased method.

Fast MBF estimations and delay maps obtained by the Bayesian approach could be a crucial clinical outcome, since an increasing number of trials (Giang et al., 2004; Schwitter et al., 2008, 2013) have shown that stress-only MBF has a better diagnostic and predictive performance than coronary flow reserve (CFR) for the detection of stenosis. In addition, the availability of this index for routine clinical use could further enable hierarchical or more complex modeling in which the MBF will still be a model parameter of paramount importance. It is beyond the scope of this paper to further investigate how fast and accurate estimations with the Bayesian method would allow more precise prior knowledge to refine the MBF initial range when fed into more complex models, which would probably also increase the accuracy. Future work could further address this hypothesis. Our *in vivo* results provided confidence in the method’s ability to improve myocardial perfusion quantification, especially in the presence of indicator arrival delays with precise pixelwise spatial information, and the estimation process showed excellent repeatability.

## Data Availability Statement

The datasets generated for this study are available on request to the corresponding authors.

## Ethics Statement

The studies involving human participants were reviewed and approved by IRBN 052019/CHUSTE. The patients/participants provided their written informed consent to participate in this study.

## Author Contributions

CD, PC, and MV were the main contributors. TB contributed by his expertise on Bayesian approach. SG contributed by providing expertise and material of acquisition sequence framework. HR contributed by providing expertise on wild bootstrap approach. M-PJ contributed by providing expertise and material for series registration. J-PV contributed by providing expertise on medical myocardial perfusion imaging. All authors contributed to the article and approved the submitted version.

## Conflict of Interest

SG and M-PJ were employed by company Siemens Medical Solutions USA, Inc. TB was employed by Olea Medical. The remaining authors declare that the research was conducted in the absence of any commercial or financial relationships that could be construed as a potential conflict of interest.

## Abbreviations

AIF: arterial input function
BAT: bolus arrival time
CAD: coronary artery disease
CFR: coronary flow reserve
CNR: contrast-to-noise ratio
FLASH: fast low-angle shot
LV: left ventricle
MBF: myocardial blood flow
P × S: permeability–surface area product
SVD: singular value decomposition
SNR: signal-to-noise ratio
TE: echo time
TR: repetition time
S_*t*_(t): signal intensity curve
S_*a*_(t): arterial input time-intensity curve
S_*N*_: normalized signal
S_0_: equilibrium signal
C_*a*_(t): arterial contrast agent input function concentration-time curve
C_*t*_(t): tissue contrast agent concentration-time curve
Ĉ_*t*_(t): time-intensity curve fit processed by the deconvolution approach
*e*^∗^: vector of C_*t*_(t) and Ĉ_*t*_(t) difference
*e*^(WB)^: set of residual-modified vectors
a_*i*_: weighting factor for the heteroscedasticity-consistent covariance matrix estimator
λ: Rademacher distribution
c^(WB)^: set of wild bootstrap concentration-time curves
C_*trep*_(t): most representative concentration-time curve of a region.
