# Supplementary material for: Direct Comparison of Bayesian and Fermi Deconvolution Approaches for Myocardial Blood Flow Quantification: In silico and Clinical Validations
Source: Front Physiol. 2021 Apr 12;12:483714. doi: 10.3389/fphys.2021.483714 (PMC8072361; doi:10.3389/fphys.2021.483714)
Supplement: Supplementary file 1 [file Table_1.DOCX]

*
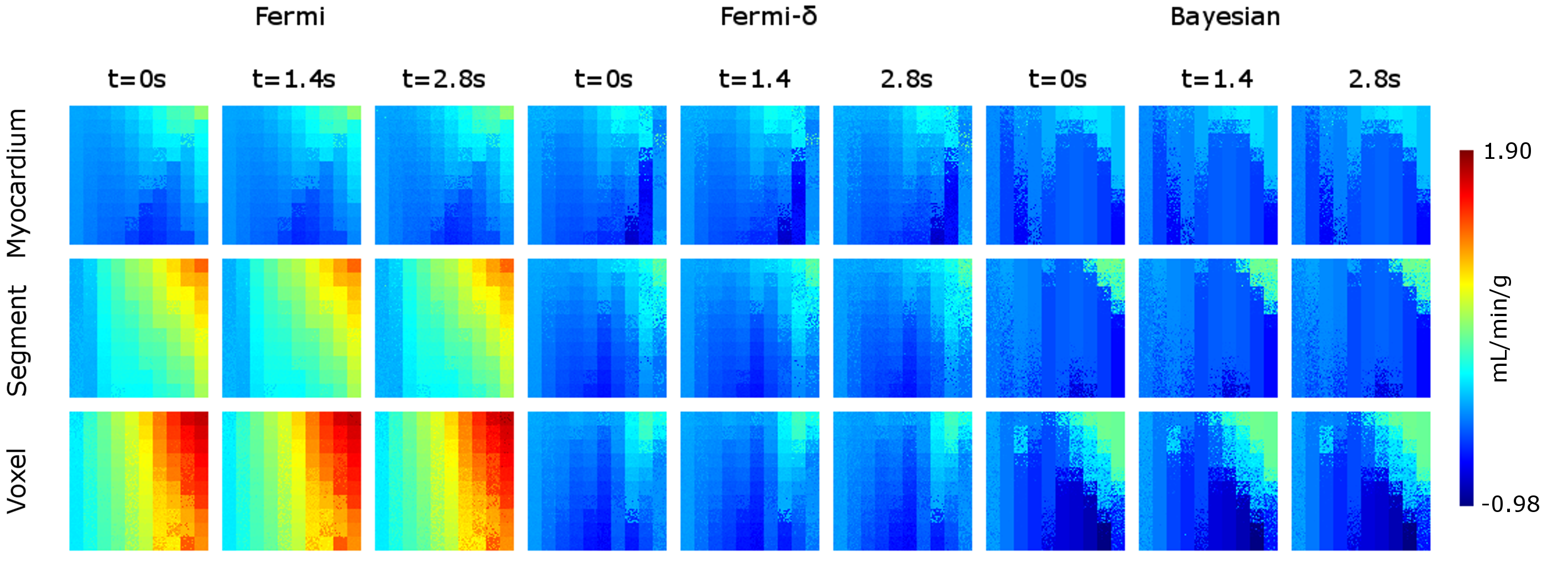
*

*Figure A1: MBF error maps calculated from estimations obtained by the three tested approaches Fermi, Fermi-δ, and Bayesian against the generated digital phantom. MBF values are given in Figure 2.*

*
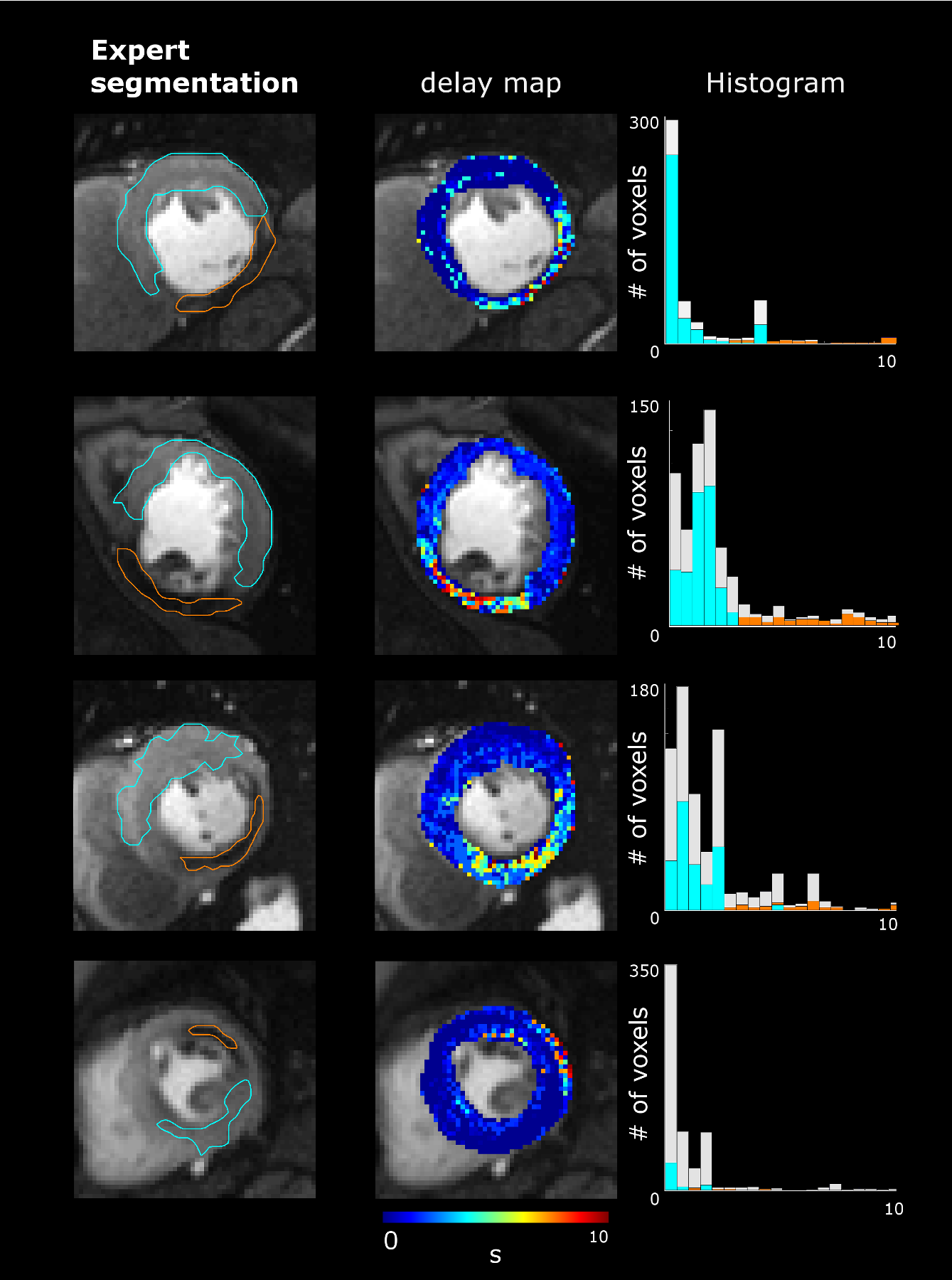
*

*Figure A2: Delay maps calculated by the Bayesian approach over the same patient slices as in Figure 7. The boundaries, defined by expert manual segmentation, delineate the normal (cyan) and abnormal (orange) regions. The colors used in the histogram indicate the proportion of voxels belonging to normal and abnormal regions, while light gray indicates the overall repartition of time delays.*
